# In Silico Strategies for Designing of Peptide Inhibitors of Oncogenic K-Ras G12V Mutant: Inhibiting Cancer Growth and Proliferation

**DOI:** 10.3390/cancers14194884

**Published:** 2022-10-06

**Authors:** Mehreen Ghufran, Haider Ali Khan, Mehran Ullah, Sabreen Ghufran, Muhammad Ayaz, Muhammad Siddiq, Syed Shams ul Hassan, Simona Bungau

**Affiliations:** 1 Department of Pathology, Medical Teaching Institution Bacha Khan Medical College (BKMC) Mardan, Mardan 23200, Pakistan; 2 Department of Biochemistry, Abdul Wali Khan University Mardan, Mardan 23200, Pakistan; 3 District Medical Officer, Sehat Sahulat Program (SSP), KPK, Mardan 23200, Pakistan; 4 District Headquarter (DHQ) Hospital Mardan, Mardan 23200, Pakistan; 5 Department of Pharmacy, Faculty of Biological Sciences, University of Malakand, Chakdara 18800, Khyber Pakhtunkhwa, Pakistan; 6 Department of Pharmacy, Abdul Wali Khan University Mardan, Mardan 23200, Pakistan; 7 Shanghai Key Laboratory for Molecular Engineering of Chiral Drugs, School of Pharmacy, Shanghai Jiao Tong University, Shanghai 200240, China; 8 Department of Natural Product Chemistry, School of Pharmacy, Shanghai Jiao Tong University, Shanghai 200240, China; 9 Department of Pharmacy, Faculty of Medicine and Pharmacy, University of Oradea, 410028 Oradea, Romania

**Keywords:** *K-Ras* G12V protein, permutations, molecular dynamics simulation, post molecular dynamics simulation

## Abstract

**Simple Summary:**

The most well-known oncogene and one with the highest rates of mutation across all cancers is K-Ras, also known as the Kirsten rat sarcoma viral oncogene homologue. It is also linked to certain cancers with a high mortality rate, including pancreatic ductal adenocarcinoma (PDAC), non-small-cell lung cancer (NSCLC), and colorectal cancer (CRC). The aim of this study was to make peptides that inhibit *K-Ras* G12V by computer-aided drug design methods. Our results showed that the top four selected peptides interact with K-Ras more strongly than the reference-peptide and can stop *K-Ras* from activating. Our binding affinity analyses demonstrated that the developed peptides can inhibit K-Ras and slow cancer growth.

**Abstract:**

Ras plays a pivotal function in cell proliferation and is an important protein in signal transduction pathways. Mutations in genes encoding the Ras protein drive the signaling cascades essential for malignant transformation, tumour angiogenesis, and metastasis and are responsible for above 30% of all human cancers. There is evidence that *N-Ras*, *K-Ras*, and *H-Ras* play significant roles in human cancer. The mutated *K-Ras* protein is typically observed in malignant growths. Mutant *K-Ras* is the most common in lung, colon, and pancreatic cancers. The purpose of this research was to create peptides that inhibit *K-Ras* G12V. The crystal structure of the mutant *K-Ras* G12V-H-REV107 complex was obtained from a protein data bank. Further, we used a residue scan approach to create unique peptides from the reference peptide (H-REV107). AMBER molecular dynamics simulations were used to test the stability of the top four proposed peptides (based on binding free energies). Our findings showed that the top four selected peptides had stronger interactions with *K-Ras* than the reference peptide and have the ability to block the activation function of *K-Ras*. Our extensive analyses of binding affinities showed that our designed peptide possesses the potential to inhibit *K-Ras* and to reduce the progression of cancer.

## 1. Introduction

The thorough examination of retroviral oncogenes obtained from the murine sarcoma viruses Harvey and Kirsten, which are derived from the genomes of rats, led to the discovery of RAS [1]. The RAS protein is encoded by three expressed genes: *Harvey-RAS* (*H-RAS*), *Kirsten-RAS* (*K-Ras*), and neuroblastoma-*RAS* (*N-RAS*). RAS is a proto-oncogene that is mutated in human cancer. One of the first cancer-initiating genes to be identified was a member of the RAS proto-oncogene family, which was discovered more than 40 years ago. *K-Ras* is the most frequently mutated RAS family member in human cancers [2]. Small GTPases known as the H, K, and NRas proteins are master controllers of numerous signaling cascades involved in a wide range of biological functions, including cell division, differentiation, cell–cell adhesion, growth, and apoptosis. The small GTPase RAS proteins function as “molecular switches” that switch back and forth between the active and inactive states. Guanine nucleotide exchange factors encourage the conversion of guanosine triphosphate (GTP) to guanosine diphosphate (GDP), which can activate these switches (GEFs; nucleotide exchange factors). GTPase-activating proteins (GAPs) encourage the hydrolysis of GTP to GDP, which results in their inactivation [3].

*K-Ras*, also known as the Kirsten rat sarcoma viral oncogene homologue, is the best-known oncogene and has the highest mutation rate among all cancers. It is also associated with a series of cancers that have a high mortality rate, such as pancreatic ductal adenocarcinoma (PDAC), non-small-cell lung cancer (NSCLC), and colorectal cancer (CRC). Cancer therapy techniques and clinical results have been transformed by the identification of tumour driver genes and the creation of targeted inhibitors [4]. RAS oncogenes are the most frequently mutated genes in human cancer, but RAS-driven tumours are not associated with those tissues where these genes are normally expressed. *K-Ras* is the most frequently mutated oncogene in humans: more than 80% of pancreatic cancers and more than 30% of colorectal and cholangial cancers and lung adenocarcinomas harbour activating mutations of *K-Ras* gene as one of the founder carcinogenic mutation in the genome. Although it can be mutated at a low percent in almost any cancer type, higher than 10% mutation rates characterizes only ovarian and endometrial cancers. *N-Ras* is the second most frequently mutated. In malignant melanoma, the mutation rate is approximately 20%, and a 10% rate characterizes colorectal cancer and hematopoietic malignancies [5,6]. *K-Ras* (Kirsten rat sarcoma virus) mutations are present in roughly one in seven of all human cancers, making it one of the main oncogenic causes of cancer in humans [7]. *H-Ras* mutations are uncommon in human malignancies, with only bladder and cervical tumours having a rate of more than 10% [8]. The RAS–RAF–MEK–ERK pathway is a conserved signaling pathway that plays vital roles in cell proliferation, survival, and differentiation. The aberrant activation of the RAS–RAF–MEK–ERK signaling pathway induces tumours [9]. *K-Ras* is an EGFR downstream effector. *K-Ras* activation is thought to promote the RAS/RAF/MEK/signaling pathway independently of EGFR activation [10]. Mutations in the above pathways’ *K-Ras*, BRAF, and PIK3CA have been found in a variety of cancers. The identification of these mutations in tumours has predictive or prognostic relevance for clinical use. Anti-EGFR medications should be given only to patients with wild-type *K-Ras* in colorectal cancer [11]. Multiple approaches have been used to identify the malignant activity of the *K-Ras* G12V mutation in various cancers such as colorectal cancer, lung cancer, and pancreatic cancer in patients and animal models [12,13,14,15,16,17,18,19]. In the case of gastric cancer, it was discovered that K-Ras G12V is a poor prognostic marker which had not previously been identified. Furthermore, this cohort is the largest in the related reports [10], which can more effectively reflect the molecular profile of southern China gastric cancer (GC) patients [10]. The G12V mutation is considered to be one of the most frequent *K-Ras* codon 12 mutations in colorectal cancer patients particularly in those with liver metastasis (ranging from 20.5% to 32.8%) [20]. With almost 44,000 new cases of *K-Ras* G12V mutant cancers diagnosed every year in the US, this condition still has a significant urgent medical need. The incidence of the *K-Ras* G12V mutation is high in several tumour histotypes; it is 6%, 10%, and 26% in NSCLC, CRC, and pancreatic cancers, respectively [21]. *K-Ras^G12D^* and *K-Ras^G12V^* are most prevalent, at 39.2 and 32.5% of all *K-Ras* mutations, respectively. The G12R mutation also occurs at high frequency in PDAC (17% of all *K-Ras* mutations) [22].

Protein–protein interactions (PPIs) are well-known as a promising class of drug targets due to their implications in a wide range of biological processes. Drug development for PPIs, on the other hand, is inevitably hampered by their flat and wide interfaces which generally lack suitable pockets for ligand binding, rendering most PPI systems “undruggable” [23]. To inhibit protein binding, the conventional method of designing peptide inhibitors is to introduce tailored oligopeptides that are modified based on several important residues along the original PPI interfaces (e.g., hot spots). It is worth noting that efforts to design peptide inhibitors have made impressive progress over the past decade. For peptide-related drug molecule rational design, the questions of how to achieve higher affinity and better synthesis diversity have also persisted for a long time. Cyclic peptides, hydrogen bond surrogates, and stapled peptides, which can improve the ADME (absorption, distribution, metabolism, and excretion) properties of peptide-based compounds, are just a few of the promising solutions that have been proposed in response to rapid advances in medicinal chemistry and chemical biology [23]. Furthermore, Walensky and associates created hydrocarbon stapled peptides based on the helical region of SOS1 that interacts with KRas [24,25]. John H. McGee et al. has created a new family of mini-proteins that block Ras-effector interactions by binding to the Ras effector domain with high affinity in vitro [26]. Mini-proteins with strong affinity for Ras were identified by the screening of naive combinatorial libraries using yeast surface display (YSD) [27].

As the most frequently mutated gene in human tumours, *Kirsten-RAS* (*K-Ras*) has been the focus of medication, according to Chang Woo Han et al.’s 2020 study [28]. Directly attacking these tumour genes was challenging due to the low drug affinity for K-Ras mutations. Work published by Han, C.W.; Jeong, M.S.; Ha, S.C.; and Jang, S.B. A h-rev107 peptide inhibits tumour growth and interacts directly with oncogenic *K-Ras* mutants. Cancers 2020, 12, 1412 reports direct interaction between *K-Ras* G12V-H-REV107, with affinity in low µM range, as determined by ITC (isothermal titration calorimetry). *K-Ras* G12V-H-REV107 (https://www.rcsb.org/structure/7c41) (accessed on 16 June 2021), an oncogenic mutant, has been published in its first crystal structure [28]. This peptide was demonstrated to engage with K-Ras G12V in the GDP-bound inactive state and to form a stable complex, preventing *K-Ras* from performing its activation function. By using a [-32P] GTP binding assay, it was demonstrated that the peptide inhibited mutant *K-Ras* targets. In a cell proliferation experiment, the H-REV107 peptide suppressed pancreatic cancer and colon cancer cell lines. Particularly, in xenotransplantation mice models, the H-REV107 peptide can inhibit pancreatic tumour growth by reducing tumour volume and weight [28]. To fully comprehend the molecular mechanism by which H-REV107 suppresses oncogenic *K-Ras* mutations, it is critical to characterize the molecular features of how H-REV107 binds *K-Ras* G12V. The biochemical mechanism by which the H-REV107 protein family is able to block the RAS signaling pathway is not currently understood. In this research, we investigated whether *K-Ras* and H-REV107 protein/peptide interact with one another directly. Immobilized variants of *K-Ras* (G12V and G12D) and the H-REV107 protein/peptide exhibited high binding affinity in surface Plasmon resonance (SPR). Binding of the H-REV107 peptide to *K-Ras* mutants demonstrated lower GTP binding affinity of *K-Ras* mutants, demonstrating that this peptide acted as a regulator of guanine nucleotide binding. Specifically, the H-REV107 peptide inhibited GTP binding to the K-Ras mutants, and this effect was seen for both K-Ras G12V and G12D. H-REV107 peptide treatment significantly suppressed the growth of pancreatic cancer and colon cancer cell lines by induction of apoptosis in a cell proliferation assay. Peptides typically have a higher working concentration of cellular action compared with small inhibitor substances. Interaction with K-Ras oncogenic mutants (G12V, G12D, G12C, G13D, and Q61H) was hypothesized to reduce KRAS mutant GTP binding affinity. K-Ras mutant targets (G12V, G12D, G12C, G13D, and Q61H) are blocked from binding to GTP by the H-REV107 peptide (65LYDVAGSDKY74). The H-REV107 peptide displayed the highest reduction in GTP binding to the K-Ras G12V mutant, among their interactions, demonstrating that it effectively suppressed the oncogenic mutant K-Ras G12V. Overall, the findings reported here will aid in the creation of innovative medications to prevent K-Ras mutations in cancer patients [28].

In this study, K-Ras G12V H-REV107 peptide (65LYDVAGSDKY74) remodeling based on in silico mutagenesis was carried out to limit K-Ras activities. Free energy calculations and molecular dynamics simulations both supported the activity. This research provides the foundation for designing peptide inhibitors that target the K-Ras G12V target to fight cancer.

## 2. Materials and Methods

### 2.1. Structure Retrieval

The protein databank (PDB code: 7c41) was used to get the crystal structure of the mutant complex K-Ras/H-REV107 (https://www.rcsb.org/structure/7c41) (accessed on 16 June 2021). This mutant GTPase K-Ras complex is a tetramer protein which means it has four similar chains, each with 174 amino acid residues, and it interacts with the H-REV107 peptide (65LYDVAGSDKY74) which has 10 residues. A ligand, ions, and water molecules are also present in the complex structure.

### 2.2. Alanine Scanning Strategy

Designing small peptide inhibitors requires an understanding of the binding interface between two interacting proteins or peptides. In the design of short peptide derivatives, the utilization of small peptides produced from the parent peptide has been extensively exploited [29,30]. Using the machine learning technique implemented in MOE [31], a similar approach was used to modify the interface residues of the reference H-REV107 peptide of the K-Ras G12V mutant and to create peptides against it. To identify the significance of each residue in the reference H-REV107 peptide that interacts with K-Ras, an in-silico alanine scanning technique was used. The dAffinity and dStability parameters in MOE’s alanine scanning module (ASM) were tracked in order to understand this tactic. The relative change in binding energy that occurs when an amino acid is converted into alanine is shown by the dAffinity and dStability values. After validation of the essential residues and selection of the hotspot residues for the residue scan to be replaced by the 19 amino acids, these scores give crucial information about the significance of a particular residue. The residue scan technique was so employed.

### 2.3. Residue Scan Strategy

The Molecular Operating Environment (MOE) software’s Residue Scan function was used to assess the dStability and dAffinity of a particular amino acid residue. The relative change in binding energies when a given amino acid was changed into another specific amino acid was demonstrated by the dStability and dAffinity scores. In order to accomplish this, the MOE residue scan module was utilized with the LowModeMD ensemble and the Unary Quadratic Optimization (UQO) parameter. A database of mutant peptides with their corresponding scores was created as a result of the residue scan. The specific mechanism of these residue scan and alanine scanning mutagenesis techniques has already been covered [32].

### 2.4. Construction of Peptides Library

Knowing the binding interface of the two interacting proteins is essential for the design of the inhibitors (peptide). In order to prevent the contact of the binding interfaces of the proteins, the peptides were created from the binding interfaces; this technique was heavily utilized in the creation of tiny peptides. The interfacial residues of the H-REV107 peptide inhibitor were modified using a similar method; the peptides were created using the MOE programme to target the K-Ras protein. By replacing the amino acid residues that were least engaged in the protein-K-Ras interaction in a ten-residue-long region of the H-REV107 peptide, as determined by the MOE suite’s Residues scanning tool, K-Ras binding peptides were designed. Five independent MD simulations were then run for the designed mutant systems and the wild-type mutant H-REV107-peptide/K-Ras complex.

### 2.5. All-Atom Molecular Dynamics Simulation

The molecular dynamic stability of the rational design peptides attached to the K-Ras target receptor and the actual mutant H-REV107/K-Ras complex was investigated using the thorough protein MD simulation approach. The top four rationally designed peptide complexes with good dStability and dAffinity values were chosen and subjected to MD simulation together with the reference peptide/K-Ras complex using the Amber 18 software [33] with the forcefield (ff14SB). Through the use of the solvate box of the Transferable inter-molecular potential with 3 points (TIP3P) water model, the top four proposed peptide/K-Ras complexes and the reference mutant H-REV107/K-Ras complex were solvated correctly. After that, the tleap was used to introduce counter-ions such as chloride or sodium ions into the solvate box, neutralizing the systems. Afterward, the neutralized complexes were subjected to energy minimization for 1000 steps using steepest descent minimization and then 500 steps using conjugate gradient minimization. After the systems had been minimized, they were heated to 300 kelvin for 50 picoseconds (ps) using the default parameters. Each solvated complex system underwent a 50-picosecond equilibration period with weak restrictions on the complex, followed by a 0.5 ns equilibration period at constant pressure and 300 K. Finally, all the equilibrated complex systems were run via a 200 ns MD simulation at constant pressure and temperature. The Langevin thermostat (1 atm, 300 K) was employed to regulate the temperature [34]. The AMBER v2018 Particle Mesh Ewald (PME) algorithm was utilized to calculate long-range interactions [35,36]. The value of 10 Å was utilized as the cutoff distance for Van der Waals and long-range electrostatic interactions. SHAKE algorithm was utilized for covalent bonding [37]. For all processes, GPU-accelerated simulation with (PMEMD.CUDA) was employed [38]. To evaluate the MD trajectories, the CPPTRAJ module in Amber v2018 was utilized. Origin Pro Lab v2018 and MOE 2019 were used to create graphical representations and do interface analysis.

### 2.6. Molecular Mechanics Generalized Born Surface Area (MMGBSA) Calculation

Using MD snapshots of binding partners recovered from explicit simulations, a conventional MMGBSA analysis was conducted and binding free energies were calculated.

(1) $$\Delta G_{\mathrm{bind}}=\Delta E_{\mathrm{MM}}+\Delta E_{\mathrm{pol}}+\Delta \mathrm{Enp}-T\Delta S$$

where E_MM_ is the energy of the entire gas phase (bond and non-bond interactions included), ΔEpol and ΔEnp are the polar and nonpolar contributions from the GB solvent models, and S is the entropy of the configuration [39,40]. Since the same snapshots are utilized for both the complex and the free receptor, the flexibility of the receptor is often not considered in such analyses. In order to take into consideration the flexibility of the binding partners, Amber GB/SA scoring (a variation of MM/GBSA) uses independent GB implicit simulation trajectories for the receptor, ligand, and the corresponding complex. S is not yet included in the Amber score of Dock 6.7 [41]. The MMPBSA/GBSA method combines continuum solvent methods with molecular mechanical energies. Here, the binding energy for the wild-type and mutant systems was determined using the Python function MMPBSA.py in Amber 18. K-Ras and the logically created peptides’ binding free energy (BFE) was calculated using the MMPBSA.py software [42]. The MM/PB(GB)SA script, which comes with AMBER and AMBER Tools, was utilized in this study to automatically carry out all the tasks required to calculate the binding free energy of a protein–protein complex using both the MM–PBSA and GBSA approaches. Using 20,000 snapshots taken along the 200 ns trajectory, the BFE was calculated. The free energy of binding was computed as follows:

$$\Delta G_{\mathrm{bind}}=\Delta \mathrm{Gcomplex}-[\Delta \mathrm{Greceptor}+\Delta \mathrm{Gligand}]$$

where the total BFE is denoted by ΔG_bind_, and the free energy of the complex, the protein, and the ligand are shown by the remaining components.

## 3. Results and Discussion

The four newly formed peptide inhibitors of the K-Ras target were discovered to influence the K-Ras activity in the current investigation by interacting with the K-Ras receptor interface and causing significant changes in the activity and structure of the K-Ras protein. K-Ras activity was significantly affected by the overall outcome.

### 3.1. The Interface Analysis of the Mutant K-Ras/H-REV107 Complex Structure

Using the PDB code, the mutant K-Ras/H-REV107 structure was retrieved (7c41). A K-Ras/H-REV107 complex is created when the 174 amino acid K-Ras protein binds to the 10 amino acid long H-REV107 peptide. The reference peptide, H-REV107, created three hydrogen bonds with the K-Ras protein, using the MOE programme. Three H-bonds were created between the Ser 17 and Ala 59 residues of the K-Ras protein and the Asp 177 and Tyr 176 residues of the reference peptide. This demonstrates that the decapeptide functions as a K-Ras inhibitor and ultimately prevented the K-Ras protein from performing its function.

### 3.2. Residue Scan to Design a Peptide Library

Using the MOE software, residue scanning was undertaken to determine the role of the H-REV107 peptide in the K-Ras receptor. Using the residue scan method, an integrated module of the Molecular Operating Environment, the interface residues of the peptide and the K-Ras protein were determined (MOE). The primary hotspot residues in the decapeptide, which was used as the reference peptide, are Tyr 176 and Asp 177, which form strong contacts with the K-Ras, while the other remaining residues were shown to be less significant at the interface to make interactions. In the first phase, we employed an alanine scanning approach to identify the best residue for fixed amino acid substitution in order to boost the binding affinity of the reference peptide. The Alanine Scan approach can aid in the selection of promising residues for mutation. All ten residues of the refpeptide were examined using the Alanine Scan method. The results revealed that only two residues had extremely high negative dAffinity (change in affinity) scores, meaning they were more crucial for interactions, whereas the other nine residues had extremely high positive values for dAffinity, meaning they were less crucial for interactions with the K-Ras receptor (wild). Alanine scanning, which calculates the relative binding affinity changes following substitution, revealed the dAffinity of each residue substitution in order to search for the most significant residues. Eight residues were preferred for substitution, according to the alanine scanning analysis, which may affect how the peptide binds. To improve the binding affinity of the reference peptide, these residues (Leu 175, Val 178, Ala 179, Gly 180, Ser 181, Asp 182, Lys 183, and Tyr 184) could be swapped out for others. Each chosen residue was substituted by one of nineteen distinct amino acids, and the binding affinities for each were determined. This residue scan analysis was observed via dAffinity, which was measured as a difference between the binding energies of the wild-type residue and the corresponding mutated amino acid residue.

Using residue scanning, ten unique peptides were produced. These ten unique mutated peptides were subjected to the per-mutations study which led to the formation of several mutant residue combinations in the peptide inhibitors. In this study, a library of peptide inhibitors (10 amino acid residues long) was theoretically created by changing the original reference sequence of the reference peptide at residues 65LYDVAGSDKY74 (original numbering was from 65 to 74 residues; new numbering given to this 10 residues long peptide is 175–184). Except for Tyr 176 and Asp 177, both of which were found to have polar interactions in the original reference peptide, every residue was changed. The research demonstrated that the MOE suite was used to construct the library of peptide per-mutants by permutation analysis. A total of 133 peptides with up to three mutations per peptide were used to generate the final peptide library (per-mutant). Peptides with one substitution and one residue difference from the reference peptide are referred to as one-point mutant peptides, whereas those with two residue differences from the reference peptide are referred to as two-point mutated peptides. Three-point mutant peptides are those that differ from the reference peptide by three residues. This residue scan study was carried out using dAffinity, which was calculated as the difference in binding energies between the wild-type residue and the matching mutant amino acid residue. The best four three-point modified peptides were chosen from a pool of 133 mutant peptides based on their Affinity and dAffinity ratings (Table 1). The lower the affinity values, the more stable the peptides will be. The negative value indicated a significant interaction.

In the current investigation, it was found that the *K-Ras* target and designed peptide had greater binding when the affinity score was lower. The biochemical study revealed that the altered residues, such as histidine, serine, and aspartate, had a substantial role in inhibiting the biological activity of the *K-Ras* target, and in this way, residue scan analysis was demonstrated to be legitimate from a literature review [43].

### 3.3. Molecular Dynamics Stability of K-RasPeptides Complexes

**Figure 1:**
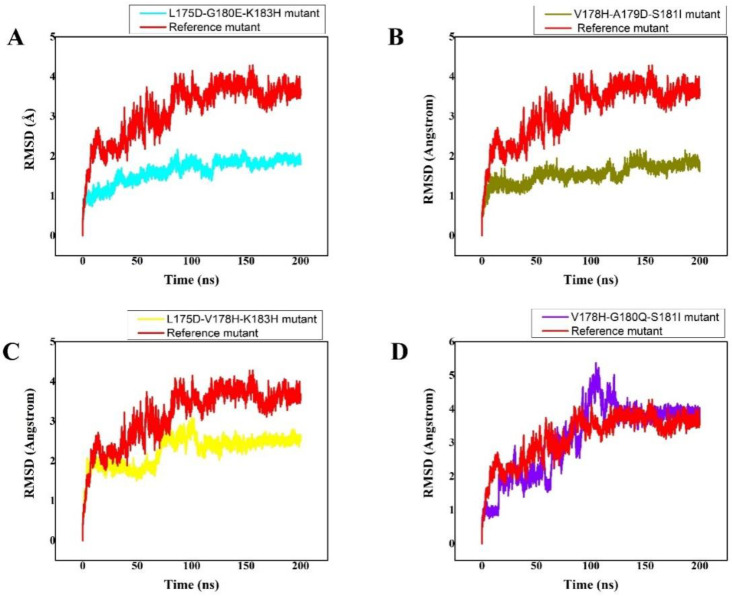
(**A**–**D**) Root mean square deviation (RMSD) of the backbone atoms of reference peptide/K-Ras mutant complex and its designed peptides/K-Ras mutant complexes: The X, Y plots show the time-dependent RMSD behaviour from the initial state to over a 200 ns trajectory. This figure shows the dynamic stability of each system as RMSD. The color scheme shows their respective trajectory. The reference peptide/K-Ras mutant complex is shown in red while the (**A**–**D**) designed peptides/K-Ras mutant complexes are shown in cyan, olive green, yellow, and purple, respectively. The *x*-axis shows time in nanoseconds while the *y*-axis shows RMSD in Angstrom.

A molecular dynamics simulation of all the complexes was conducted to look at the dynamic properties of these developed peptides in contact with K-Ras protein. To measure each system’s stability, the root mean square deviation (RMSD) was determined as a function of time. The reference peptide/MD complex’s simulation findings revealed that the RMSD graph of the route to their initial structure varied from 0.0 to 3.9. (Figure 1). The RMSD graph fluctuated up to 150 ns of MD simulation, and the value increased to 4, but after 150 ns, as well as from 150 ns to 200 ns of MD simulation, the fluctuation was not seen, and then more fluctuations were seen from 150 ns to 180 ns, indicating that the complex attained its stability after 180 ns. A most stable and energy-minimized conformer was achieved after 200 ns of simulation; this can be utilised as a standard (control) for comparison with the generated peptides. However, compared with the wild type, the (S. No 1) L175D-G180E-K183H peptide/K-Ras system showed the least RMSD and did not exhibit any significant fluctuations. The L175D-G180E-K183H peptide/K-Ras had an average RMSD of 1.8 Å. Additionally, compared with the wild complex, all of the designed peptides remained more stable. Between 0 and 100 ns, the RMSD fluctuated somewhat but stayed between 1 and 2 Å. After 100 ns, the system returned to equilibrium with 1.8 Å of RMSD, and after 150 ns, the system became more stable (Figure 1A). The average RMSD for the (S. No 2) V178H-A179D-S181I peptide/K-Ras system was 1.8 Å, and while minor fluctuations were occasionally seen, the system was generally stable from 0 to 200 ns (Figure 1B). The (S. No 3) L175D-V178H-K183H system, on the other hand, also maintained stability, with fluctuations ranging from 55 to 100 ns. The L175D-V178H-K183H system’s stated average RMSD was 2.5 Å. After 150 ns of molecular dynamic simulation, the system became more stable (Figure 1C). The peptide complex of (S. No 4) V178H-G180Q-S181I fluctuated more (from 0 to 5 Å of RMSD) between 0 and 125 ns. The system eventually reached stability with fewer variations between 125 and 200 ns after 125 ns (Figure 1D). These findings indicated that the mutant designed peptides bound more tightly than the wild type and favoured the stability of the entire complex since they demonstrated that the mutant systems remained more stable during the simulation than the wild type. All the RMSD graphs are given in Figure 1.

### 3.4. Residual Flexibility of the Complexes

**Figure 2:**
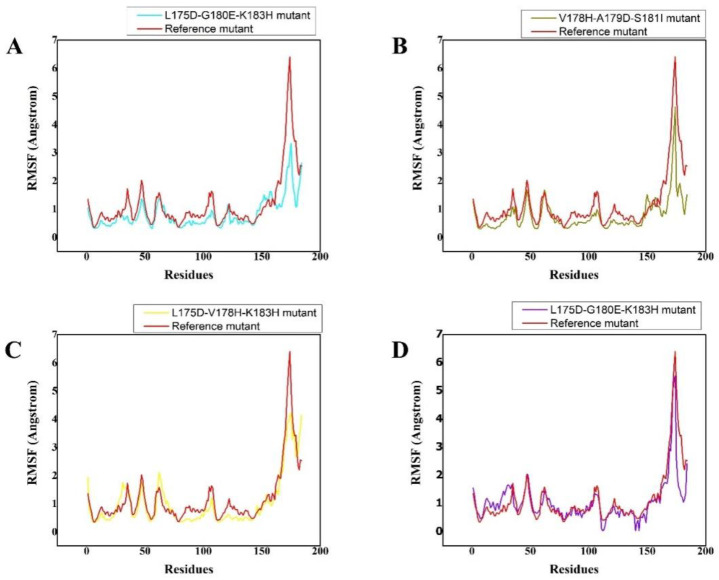
(**A**–**D**) The RMSF of reference peptide/K-Ras mutant complex (red) and its derivative peptide/K-Ras mutant complexes (cyan, olive green, yellow, and purple, respectively). The X, Y plots show time-dependent RMSF behavior from the initial state to over a 200 ns trajectory of K-Ras in complex with the respectively designed peptide. The different colour scheme shows the differently designed peptides and their respective trajectories as well as the residual flexibility of each system. The *x*-axis shows the number of residues while the *y*-axis shows RMSF in Angstrom.

The RMSF plots demonstrated that substitution had a distinct impact on the flexibility of the residues in each synthesised peptide. The wild type demonstrated relatively high residual flexibility, as seen in Figure 2, which is consistent with the RMSD values. Because the wild type was observed to be relatively unstable, its residual flexibility remained larger than that of the others. The other systems demonstrated a more similar pattern of residual flexibility. The variations rose at the substitution position in the V178H-G180Q-S181I peptide complex, whereas the remaining complexes showed decreasing fluctuations. These findings showed that the chosen peptides bound to the protein target more efficiently than the wild type and influenced residual dynamics by stabilising flexibility in comparison with the wild type. Figure 2 depicts the versatility of each system in a distinct colour.

### 3.5. Hydrogen Bonds

**Figure 3:**
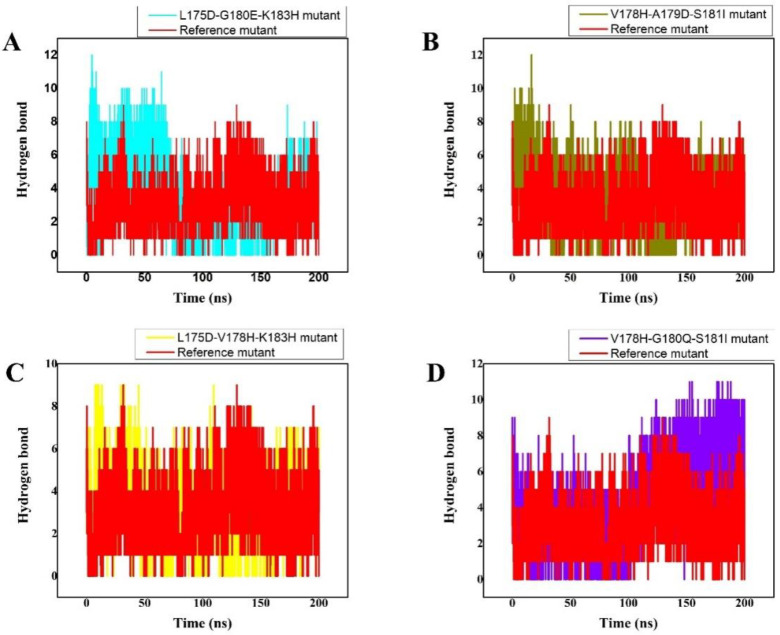
(**A**–**D**) The total number of Hydrogen bonds over 200 ns MD trajectory, the *x*-axis shows the time duration in nano second while the *y*-axis represents the number of hydrogen bonds. The total number of intermolecular H-bond interactions between K-Ras and the peptide variants. The different colours show the different designed peptides/K-Ras mutant complexes while the reference peptide/K-Ras mutant is shown in red.

The number of generated hydrogen bonds in all systems was calculated to provide a more precise examination at the atomic level. The geometric requirements for a hydrogen bond are as follows: the hydrogen-donor-acceptor angle must be ≤ to 30°, and the donor-acceptor distance must be ≤ to 0.35 nm. If these two conditions are met simultaneously, a hydrogen bond exists. Hydrogen bonds are essential for the preservation of the secondary structure (-helices) of peptides and proteins [44,45]. The results of the time-dependent hydrogen bond study showed that all ten of the proposed peptide derivatives exhibited the strongest and best hydrogen-bonding networks with the K-Ras protein (Figure 3). It was seen that all of the constructed peptide/complex systems contain many hydrogen bonds compared with the wild-type complex, for example, the L175D-G180E-K183H peptide/K-Ras complex (Figure 3A). Our study’s findings suggest that the four developed peptides have strong, exceptional K-Ras binding capacity and may function as strong peptide inhibitors.

### 3.6. Radius of Gyration

**Figure 4:**
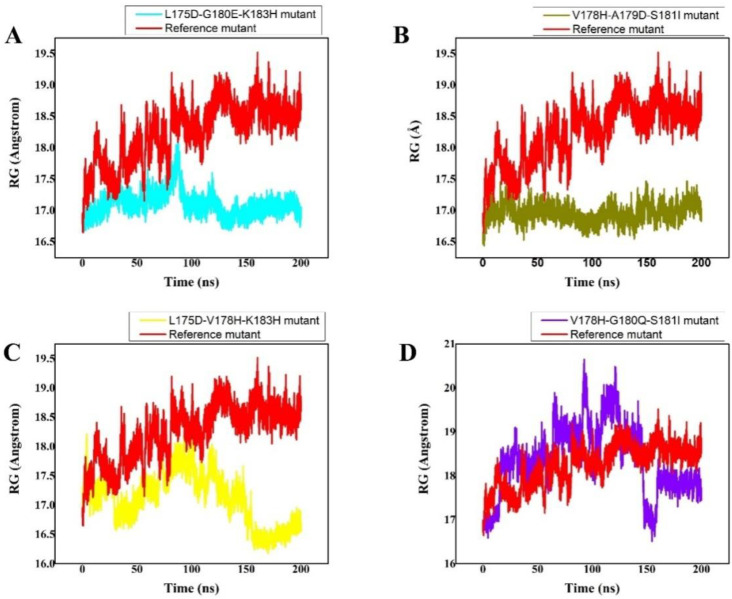
(**A**–**D**) RG (radius of gyration) of all the systems was calculated from the 0–200 ns MD simulations trajectory. The *x*-axis shows the number of frames and the *y*-axis shows RG in Angstrom. This figure represents the superposed plot for radius of gyration (Rg) in reference peptide and designed peptides mutants in complex with the K-Ras mutant. The reference peptide/K-Ras mutant complex is shown in red while the (**A**) (S. No 1) L175D-G180E-K183H peptide/K-Ras system, (**B**) (S. No 2) V178H-A179D-S181I peptide/K-Ras system, (**C**) (S. No 3) L175D-V178H-K183H/K-Ras system, (**D**) (S. No 4) V178H-G180Q-S181I/K-Ras complex are shown in cyan, olive green, yellow, and purple, respectively.

The size and compactness of the protein molecules are determined by the radius of gyration (Rg) [46]. Here, the Rg calculation was carried out to comprehend how the peptides maintained their physical integrity with the protein target throughout the simulation. The stability of the system is also determined by its compactness. The wild-type system had substantially converged Rg, as illustrated in Figure 4. Up until 40 ns of md simulation time, the Rg value was 17.5 ns; however, once this time had passed, the Rg of the wild type abruptly converged, and the Rg value rose to 18.5 ns. From 120 ns to 200 ns of time and strong fluctuations, the Rg value maintained 18.5. This demonstrates how unstable the wild-type system is; the peptide was likely released, and water entered the active pocket, decreasing the peptide binding. However, the L175D-G180E-K183H peptide/K-Ras system’s Rg remained constant, and the system displayed greater compactness. With an Rg value of 18 Å, slightly greater fluctuations were seen at 80 ns. Later, at 200 ns of simulation, the Rg value dropped to 16.8 Å, but generally, the system remained extremely compact, and the average Rg value was reported to be 16.8 Å (Figure 4A). Comparatively speaking, the V178H-A179D-S181I peptide/K-Ras system was less bulky than the wild type and L175D-G180E-K183H peptide/K-Ras. For the V178H-A179D-S181I peptide/K-Ras, the average Rg value was 16.9 Å (Figure 4B). The reduced Rg value demonstrates the peptide’s strong binding to the receptor. Between 150 and 200 ns, the L175D-V178H-K183H system remained compact, while between 0 and 150 ns, there were greater variations and unstable behaviour. For the L175D-V178H-K183H system, the average Rg was 16.9 Å (Figure 4C). Additionally, it was seen that the Rg value for the V178H-G180Q-S181I was 17.5 Å (Figure 4D). Except at the 150 ns mark, major fluctuations were seen throughout the entire experiment. In comparison to the wild-type system, the system had stabilised and was quite compact after 150 nanoseconds. These outcomes demonstrated that the planned peptides maintained their structural integrity with the active site residues during the course of the simulation. According to these findings, the designed peptides effectively bound to and inhibited K-Ras protein. Figure contains the Rg(s) for every system.

### 3.7. Binding Free Energy Calculation

The binding free energies of all the complexes have been precisely predicted using a widely known method called Molecular Mechanics Generalized Born Surface Area (MMGBSA). The binding affinity between interacting molecules is determined by the Gibbs free energy [29,30]. The table includes the total binding free energy of all the systems as well as the VDWAALS, electrostatic energy, polar solvation, solvent accessible surface area, and other energy terms. These findings strongly imply that the engineered peptides had greater potential for inhibition than the wild-type reference peptide. The contribution of the altered residues greatly raised the electrostatic and VDWAALS energies, which ultimately raised the total binding energy. Table 2 provides the total binding energy as well as all of the associated terms.

### 3.8. Interaction Analysis of the Designed Peptides with the K-Ras Protein

**Figure 5:**
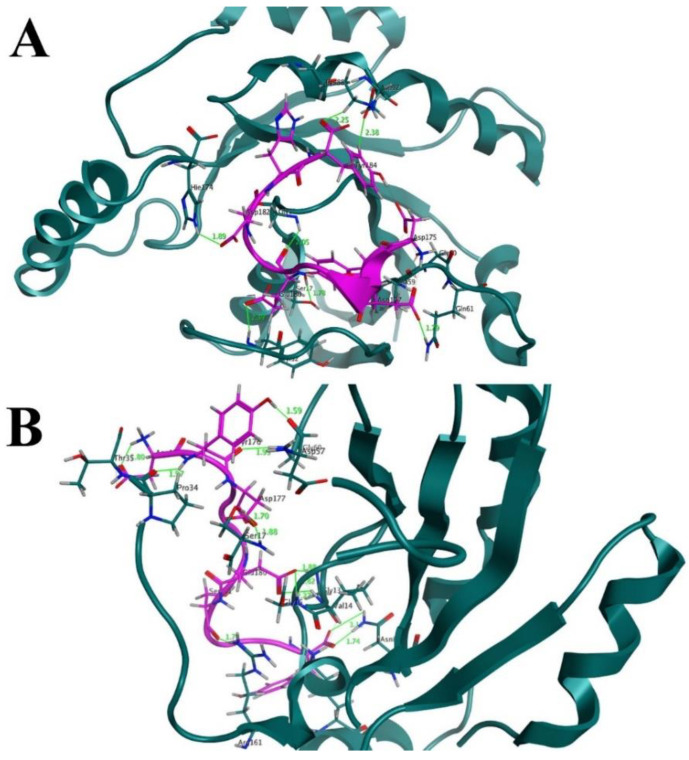
(**A**) Before MD simulation, the binding interface revealed that the designed peptide, such as (S. No 1) L175D-G180E-K183H, formed 7 hydrogen bonds with the active residues of K-Ras receptor. A hydrogen bond could be seen as a solid line in green. (**B**) After 200 ns of MD simulation, the binding interface showed that the number of hydrogen bonds increased to 12 H-bonds between the residues of (S. No 1) L175D-G180E-K183H peptide (designed peptide) and residues of K-Ras mutant protein.

The H-REV107 peptide is linked to the K-Ras active residues in the 3D crystal structure. The results of the time-dependent hydrogen bond study showed that all four of the proposed peptide derivatives exhibited the strongest and best hydrogen-bonding networks with the K-Ras protein (Figure 3). It was seen that all of the constructed peptide/complex systems contain several hydrogen bonds compared with the wild-type complex, for example, the designed peptide/complex L175D-G180E-K183H. (Figure 5B). Our study’s findings suggest that the four developed peptides have strong, exceptional K-Ras binding capacity and may function as strong peptide inhibitors. In the near future we will design further experimental studies including the in vitro binding assays to K-Ras (using ITC, SPR, MST, etc.) to further validate the potential use of these peptide inhibitors in cancer.

## 4. Conclusions

As a result of these findings, a new paradigm for the creation of peptide inhibitors for the K-Ras protein was identified. The behaviour and inhibitory qualities of the suggested mutant peptides were confirmed by using a lengthy computational process which included basic molecular modelling and free energy calculations. Our extensive examinations of binding affinities revealed that our created peptide has the potential to interfere with the K-Ras protein and will lessen tumour angiogenesis, metastasis, cell proliferation, and malignant transformation. The experimental and clinical validity of these peptides to inhibit K-Ras is clearly supported by our analysis.

## Figures and Tables

**Table 1:** Peptide design by substituting residues in “LYDVAGSDKY”.

S. No	Peptide Sequence	Affinity (kcal/mol)	dAffinity (kcal/mol)
1	DYDVAESDHY	−11.4881	−1.1435
2	LYDHDGIDKY	−11.0227	−1.2644
3	DYDHAGSDHY	−10.9856	−1.0907
4	LYDHAQIDKY	−10.6353	−0.4426
WT	LYDVAGSDKY	−9.5301	0

Mutated residues are shown in red.

**Table 2:** The MMGBSA Binding free energy (kcal/mol) calculation for refpeptide/*K-Ras* complex and its variants (1–4 designed peptide/complex).

No#	Peptide Sequence	VDWAALS	ESURF	EGB	EEL	Delta Total Binding Free Energies
1	DYDVAESDHY	−86.0276	−11.5218	77.7663	−39.5265	−59.3096
2	LYDHDGIDKY	−81.6682	−10.6545	−35.5413	75.1207	−52.7433
3	DYDHAGSDHY	−77.1634	−9.6102	−103.8830	147.1721	−43.4844
4	LYDHAQIDKY	−62.7196	−8.9737	−159.3055	196.6481	−34.3507
WT	LYDVAGSDKY	−62.9248	−9.7345	54.2766	−12.8694	−31.2521

WT = Wild Type, VDWAALS = van der Waals contribution from MM, EEL = electrostatic energy as calculated by the MM force field, EGB = the electrostatic contribution to the solvation free energy calculated by GB, and ESURF = surface areas energy. Final estimated total binding free energy calculated from the terms above (kCal/mol). Mutated residues are shown in red.

## Data Availability

The data presented in this study are available on request from the corresponding author.
